# Protozoan infections are under-recognized in Swedish patients with gastrointestinal symptoms

**DOI:** 10.1007/s10096-020-03974-w

**Published:** 2020-07-07

**Authors:** Jessica Ögren, Olaf Dienus, Jessica Beser, Anna J. Henningsson, Andreas Matussek

**Affiliations:** 1 Clinical Microbiology, Region Jönköping County, Sweden and Department of Health, Medicine and Caring, Linköping University, Linköping, Sweden; 2 Division of Clinical Microbiology, Department of Laboratory Medicine, Karolinska Institutet, Stockholm, Sweden; 3 Department of Microbiology, Public Health Agency of Sweden, Solna, Sweden; 4 Division of Laboratory Medicine, Oslo University Hospital, Oslo, Norway

**Keywords:** Gastroenteritis, Diagnostics, *Entamoeba histolytica*, Cryptosporidium, *Cyclospora cayetanensis*, *Giardia intestinalis*

## Abstract

In acute gastroenteritis (GE), identification of the infectious agent is important for patient management and surveillance. The prevalence of GE caused by protozoa may be underestimated in Swedish patients. The purpose was to compare the prevalence of *E. histolytica*, *Cryptosporidium* spp., *G. intestinalis*, and *C. cayetanensis* in samples from patients where the clinician had requested testing for gastrointestinal parasites only (*n* = 758) to where testing for bacterial GE only (*n* = 803) or where both parasite and bacterial testing (*n* = 1259) was requested and a healthy control group (*n* = 197). This prospective cohort study was conducted in Region Jönköping County, Sweden (October 2018–March 2019). Fecal samples were analyzed with microscopy and real-time PCR. *Cryptosporidium* spp. was detected in 16 patients in the bacterial GE group and in 13 in the both bacterial and parasite group; no cases were detected in the group were only parasite infection was suspected. *C. cayetanensis* was detected in two patients in the bacterial GE group. One case of *E. histolytica* was detected in the bacterial group and one in the both bacterial and parasite group. *G. intestinalis* was detected in 14 patients in the parasite only group, 12 in the both parasite and bacterial group, three in the bacterial GE group, and one in the control group. Diarrhea caused by protozoa, especially Cryptosporidium was under–recognized by clinicians and is likely more common than hitherto estimated in Sweden. A more symptom-based diagnostic algorithm may increase detection and knowledge about protozoan infections.

## Introduction

In cases of acute gastroenteritis (GE), identification of the infectious agent can be of importance for patient management and for surveillance leading to early discovery of potential outbreaks that can be harmful for individuals and the community. The prevalence of the diarrhea-causing intestinal protozoa such as *Giardia intestinalis* (*G. intestinalis*), *Cryptosporidium* spp, *Cyclospora cayetanensis* (*C. cayetanensis*), and *Entamoeba histolytica* (*E. histolytica*) in a low-risk country like Sweden is not well understood, and several factors like methodology, geographical setting, population, and testing algorithms are likely to influence the data [1–4].

*Cryptosporidium* spp*.* are pathogenic intestinal parasites in humans and animals worldwide. *Cryptosporidium parvum* (*C. parvum*) and *Cryptosporidium hominis* (*C. hominis*) are the most common species, but human infection can be caused by a large number of species [5]. An infection with Cryptosporidium can cause watery diarrhea and abdominal pain, sometimes fever and vomiting. Cryptosporidiosis lasts longer (usually more than 10 days) than most episodes of gastroenteritis caused by viruses and bacteria [2, 4]. Cryptosporidium infection can lead to serious illness with prolonged diarrhea and extra-intestinal manifestations in immunocompromised patients [2]. One of the largest waterborne outbreaks in Europe of Cryptosporidium was discovered in Sweden in 2010 [6, 7], and the Nordic countries have a known high rate of foodborne cryptosporidiosis [8]. The use of real-time PCR in laboratory diagnostic of protozoan infections in Sweden has increased during the last 5–10 years, and is now available in almost all regions. The total detection in Sweden has increased from 224 (2.32 per 100,000 inhabitants, range 0–17.59 cases/100000 inhabitants) cases annually in 2013 to 779 (7.69 per 100,000 inhabitants, range 0.35–37.55 cases/100,000 inhabitants) cases in 2017 [9], and the increase is likely due to more sensitive diagnostic methods and increased awareness [4]. An increase in detected cases of cryptosporidiosis has also been seen in other parts of Europe [10].

*Giardia intestinalis* is found worldwide and is the most commonly reported protozoa causing GE in Sweden. *G. intestinalis* causes a wide range of symptoms from none to severe diarrhea [11, 12]. Most cases detected in Sweden are infected abroad. The number of detected cases in Sweden in total has not increased during the last 5 years; following the introduction of real-time PCR for screening, there were 1253 (12.6 cases/100,000 inhabitants, range 0–39.6 cases/100,000 inhabitants) cases reported in 2013 and 1143 (11.3 cases per 100,000 inhabitants, range 6.8–23.4 cases/100,000 population) in 2017. The incidence varies some over the years (from 1100 to 1400), and the same variation has been shown previously. *G. intestinalis* is most common in children aged 1–10 years [9].

*Entamoeba histolytica* is uncommon in Sweden, and patients are usually infected abroad. Diagnosis is important since the parasite can lead to severe illness [13, 14]. It is important to differentiate between *E. histolytica* and other apathogenic species of Entamoeba. Reported cases of *E. histolytica* have decreased from 164 to 46 between 2013 and 2017 [9], due to the introduction of real-time PCR which in contrast to microscopy reliably can distinguish between pathogenic and apathogenic *Entamoeba* spp. [13, 15].

*Cyclospora cayetanensis* is an intestinal parasite that has caused major outbreaks in the USA, Canada, and Mexico in recent years [16]. The incidence in Sweden is unknown because the infection is not subject to notification. A recent survey by the Public Health Agency of Sweden (not published) showed that only a few laboratories detect *C. cayetanensis* and almost all use microscopy for detection. The parasite can cause watery diarrhea, fever, muscle aches, weight loss, and long-term complications such as reactive arthritis [17].

It has been shown that, among clinicians, the knowledge of protozoan infections is low and may result in underdiagnosis [1, 18, 19]. In Sweden, no national guidelines are available regarding the referral of samples for diarrhea-causing protozoa testing. In the two regions in Sweden with the highest incidence of Cryptosporidiosis, a regimen has been implemented that all fecal samples from patients with diarrhea are routinely screened with molecular methods for most diarrhea-causing protozoa (together with bacterial and viral agents) regardless of type of diarrhea and region of exposure (domestic or travel-related). Those two regions had an incidence of 37.55 and 26.8 cases/100,000 inhabitants, respectively in 2017 [9]. The mean incidence in the other 19 regions was 5.1 cases/100,000 inhabitants in 2017, and in those regions, testing for diarrhea-causing protozoa is mostly done (with molecular methods but some use microscopy) on special request upon either parasite infection in general or specific Cryptosporidium suspicion (often immunosuppression). It is therefore possible that otherwise healthy individuals with acute diarrhea without a history of recent travelling are not tested for diarrhea-causing protozoa on a routine basis and that infections are missed due to lack of testing [2].

In this prospective study, we wanted to investigate the occurrence of *Cryptosporidium* spp., *G. intestinalis*, *E. histolytica*, and *C. cayetanensis* in fecal samples from patients presenting with gastrointestinal symptoms in Region Jönköping County, Sweden. We compared the prevalence of the protozoa in samples from patients where the clinician had requested testing for parasites only compared to where testing for bacterial gastroenteritis only or where both parasite and bacterial testing was requested. Both microscopy and multiplex real-time PCR were used for diagnostics. Cases of *Cryptosporidium* spp. were species identified and subtyped at the Public Health Agency of Sweden.

## Methods

### Study design

This prospective cohort study was conducted in Region Jönköping County, Sweden. Consecutive first fecal samples from patients suspected of having bacterial and/or parasite-caused GE between October 2018 and March 2019 were included. Patients were divided into three groups. Results were reported to the requesting clinician both when testing was requested and when it was not. Samples from blood donors were included as healthy controls. No results were reported for the control group.

### Sample collection and classification

Bacterial and parasite group (BP): Patients where the requesting clinician suspected both bacterial and parasite-caused GE (samples for both bacterial GE and parasite examination). Samples were collected in both Copan Liquid Amies Elution Swab (eSwab) (Copan, Italy) and sodium acetate acetic acid formaldehyde (SAF)-fixative. SAF-fixed samples were routinely analyzed with microscopy for cysts, oocysts, trophozoites, and ova/larvae; and samples in eSwab were analyzed using routine culture methods for detection of *Campylobacter* spp., *Salmonella* spp., *Yersinia enterocolitica* (*Y. enterocolitica*), and *Shigella* spp. In addition, real-time PCR for *G. intestinalis*, *E. histolytica*, *Cryptosporidium* spp., and *C. cayetanensis* was done on both the eSwab sample and the SAF-fixed sample.

Bacterial group (B): Patients where only bacterial GE was suspected by the requesting clinician. Samples were collected in eSwab and analyzed using routine culture methods for detection of *Campylobacter* spp., *Salmonella* spp., *Y. enterocolitica*, and *Shigella* spp. Samples were in addition analyzed with real-time PCR for *G. intestinalis*, *E. histolytica*, *Cryptosporidium* spp., and *C. cayetanensis*. Positive samples were analyzed with microscopy for verification.

Parasite group (P): Patients where only parasite-caused GE was suspected by the requesting clinician. Samples were collected in SAF-fixative. Samples were routinely analyzed with microscopy for cysts, oocysts, trophozoites, and ova/larvae and in addition with real-time PCR for *G. intestinalis*, *E. histolytica*, *Cryptosporidium* spp., and *C. cayetanensis.*

Control group: As healthy control subjects, blood donors were asked to submit a fecal sample. Data of age and sex were collected using a questionnaire. They were asked adjacent to giving blood and received a package with a sample tube (eSwab) and instructions on how to take the test at home and then send it to the laboratory. Samples were analyzed with real-time PCR for *G. intestinalis*, *E. histolytica*, *Cryptosporidium* spp., and *C. cayetanensis*. Positive samples were analyzed with microscopy for verification.

### Microscopy

The method used has been previously described in detail [20], but briefly, SAF-fixed fecal samples were homogenized, diluted with NaCl (0.9%) containing triton-X, and then filtered through a metal sieve. About 7 mL was transferred to a centrifuge tube; ethyl acetate was added, and the tubes were put in a head-over shaker followed by centrifugation. The formed plug of debris was removed and 1 mL of NaCl (0.9%) was added to the sediment. The sediment and one drop of the remaining 3 mL of the filtered fecal suspension were analyzed (× 400) for cysts, trophozoites, oocysts, ova, and larvae. Samples from patients with a history of diarrhea, fever, vomiting, muscle pain, animal contact, or immunosuppression or when an outbreak was suspected or with suspected wet mount findings were stained with modified Ziehl-Neelsen (Z-N) for detection and verification of *Cryptosporidium* spp..

Microscopy was done with a Nikon eclipse 50i microscope (Nikon instruments Europe B.V, The Netherlands).

Microscopy verification of positive results with real-time PCR from group B and the blood donor group was done on wet mounts and Z-N stained smears from eSwab.

The PCR results were not available for the microscopist. After analysis, discrepant results for real-time PCR and microscopy were re-evaluated with both methods.

### DNA extraction

The SAF-fixed samples were homogenized and filtered as described for microscopy. Thereafter, 1 mL was centrifuged for 1 min at 10000*g*. The supernatant was discarded, and the sediment was suspended in 1 ml of phosphate-buffered saline **(**PBS) and then centrifuged for 1 min at 10000*g*. The supernatant was discarded, and the sediment was re-suspended in 280 μL of AL buffer (Qiagen, Hilden, Germany) and 20 μL of proteinase K (Qiagen).

About 300 μL of samples in eSwab was transferred with a pipette to a micro centrifuge tube containing 280 μL of AL buffer (Qiagen) and 20 μL of proteinase K (Qiagen).

The suspensions from both SAF-fixed and eSwab were then incubated at 56 °C for 1 h with gentle agitation followed by treatment for 1 h in − 80 °C, and was then incubated at 98 °C with gentle agitation for 20 min. Finally, the material was centrifuged for 1 min at 10000*g*, and the supernatants were transferred to new tubes and stored in − 20 °C prior to extraction [20]. A known positive fecal sample was included in each run.

SAF-fixed fecal samples and fecal samples from eSwab were subjected DNA extraction protocol, done with a MagNA Pure LC DNA isolation kit III, in a MagNA pure 32 instrument (Roche Diagnostics GmbH, Mannheim, Germany) according to the manufacturer’s instructions. The elution volume was 200 μL.

### Multiplex real-time PCR

Real-time PCR was done with a LightCycler 480 II instrument (Roche Diagnostics GmbH) according to a previous publication [20] with addition of *C. cayetanensis* according to Verweij et al. [21] with modifications by Qvarnstrom et al. [22] correcting a missing base in the reverse primer sequence to 5′-AAT GCC ACG GTA GGC CAA TA-3′ (Tib Molbiol, Berlin, Germany). Thermal cycling protocol was: 95 °C for 5 min followed by 50 cycles of 95 °C for 5 s, 60 °C for 15 s, and 72 °C for 15 s. Two PCR mixtures were used; the first included primer and probes specific for *E. histolytica* and *C. cayetanensis*, and the second specific for *Cryptosporidium* spp., *G. intestinalis*, and the PCR inhibition control Phocine herpesvirus (PhHV). Template volume was 5 μL in a total volume of 25 μL which include LightCycler 480 Probes Master (Roche Diagnostics GmbH) and additionally 2 μL 25 mM MgCl_2_.

### Cryptosporidium molecular typing

DNA from fecal samples was extracted using the magLEAD 12gC instrument supplied with magDEA DX MV reagents (Precision System Science Co Ltd., Chiba, Japan). All extractions were performed according to manufacturer’s instruction. Prior to extraction, oocyst disruption was done by bead beating using a Bullet Blender (Techtum, Sweden). Identification of *Cryptosporidium* species was done by amplification of the small subunit rRNA (ssu rRNA) gene by PCR, followed by bi-directional sequencing of the PCR amplicons [23, 24]. Subtyping was done by amplification of the 60 kDa glycoprotein (gp60) genes followed by bi-directional sequencing of the PCR products [25]. Editing and analysis of sequences were done using CLC Main Workbench (Qiagen, Aarhus, Denmark, version 6.9.1). Obtained sequences were compared with isolates in the GenBank database using the Basic Local Alignment Search Tool (BLAST; NCBI www.ncbi.nlm.nih.gov/blast/BLAST.cgi).

### Statistics

A chi^2^ test was done to compare detection rates of protozoa in different groups and to compare sex distribution between groups. A Kruskal-Wallis test was done to compare age distribution between the patient groups. Analysis was done using Statistica 13.5.0.17 (TIBCO software Inc.). A significance level of < 0.05 was used.

## Results

**Fig. 1:**
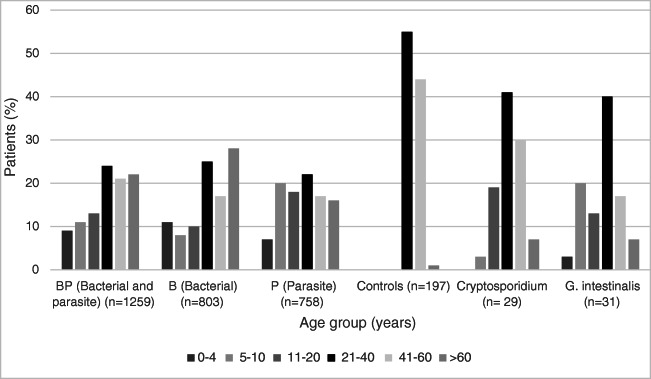
Age distribution in percentage in the different patient groups, control group and in patients positive for Cryptosporidium and *G. intestinalis*

In total, 1259 patients were included in group BP, 803 in group B, 758 in group P, and 197 in the control group. The age distribution is shown in Fig. 1. In group BP, there was 54% female and 46% male; in group B, it was 57% female and 43% male; in group P, it was 55% female and 45% male, and in the control group, it was 44% female and 56% male. There was no difference between sex in the patient groups (*p* = 0.4) but there was a statistically significant difference between the patient groups and the control group (*p* > 0.00). There was no statistically significant difference in age distribution between the different patient groups (*p* = 0.35).

The detection of *Cryptosporidium* spp., *E. histolytica*, *G. intestinalis*, and *C. cayetanensis* is shown in Table 1. The culture results for *Shigella* spp., *Salmonella* spp., *Campylocter jejuni/coli*, and *Y. enterocolitica* for patients in groups B and BP are also shown in Table 1. There was a statistically significantly higher detection rate of Cryptosporidium in group B compared to the other groups (*p* = 0.02). There was a significantly higher detection of *G. intestinalis* in groups P and BP compared to group B (*p* > 0.00). There was no statistically significance in detection of *G. intestinalis* between all the patient groups and the control group (*p* = 0.5).

**Table 1:** Detection of protozoa and bacteria in the different study groups

	BP (Bacterial and parasite GE) *n* (%)	B (Bacterial GE) *n* (%)	P (parasite GE) *n* (%)	Control group *n* (%)	Statistical difference (*p* value)
*Entamoeba histolytica*	1 (0.08)	1 (0.1)	Nd	Nd	Na
*Giardia intestinalis*	12 (0.95)	3 (0.4)	14 (1.8)	1 (0.5)	> 0.00
*Cryptosporidium* spp	13 (1.0)	16 (2.0)	Nd	Nd	0.02
*Cyclospora cayetanensis*	Nd	2 (0.28)	Nd	Nd	Na
*Campylobacter jejuni/coli*	40 (3.1)	50 (7.1)	Na	Na	
*Salmonella* spp	15 (1.2)	13 (1.8)	Na	Na	
*Shigella* spp	3 (0.2)	3 (0.3)	Na	Na	
*Yersinia enterocolitica*	4 (0.3)	Nd	Na	Na	

*Nd* not detected, *Na* not analyzed

One patient had a co-infection of *Salmonella* spp. and Cryptosporidium; two had both *Campylobacter* spp*.* and *Salmonella* spp., and one had *E. histolytica* and *G. intestinalis*.

Two of the 13 Cryptosporidium cases in the BP group were negative in real-time PCR but positive in microscopy. One was typed to *Cryptosporidium canis* (*C. canis*) and the other *C. hominis* IaA23R4. All of the remaining 11 Cryptosporidium in BP group was positive in both microscopy and real-time PCR. All Cryptosporidium positive (real-time PCR) in group B was verified by microscopy. There was no statistically significant sensitivity difference in the detection of parasites between microscopy and real-time PCR (*p* = 0.8).

Two of the three PCR positive *G. intestinalis* in the B group could be verified with microscopy, and the one positive sample in the blood donor group was verified with microscopy. One *G. intestinalis* was positive in PCR but negative in microscopy in group P. There was no difference between microscopy and PCR in the BP group.

The *C. cayetanensis*-positive samples by real-time PCR in group B were verified with microscopy. Suspected origin of infection was not given.

The patient with *E. histolytica* in the BP group was positive in microscopy for *E. histolytica/E. dispar* and positive for *E. histolytica* in real-time PCR. The PCR-positive *E. histolytica* sample in the B group was negative in verification microscopy but verified in another sample submitted later in SAF-fixative, which contained trophozoites of Entamoeba and was positive for *E. histolytica* in real-time PCR. Both patients with *E. histolytica* had recently been travelling in India.

**Fig. 2:**
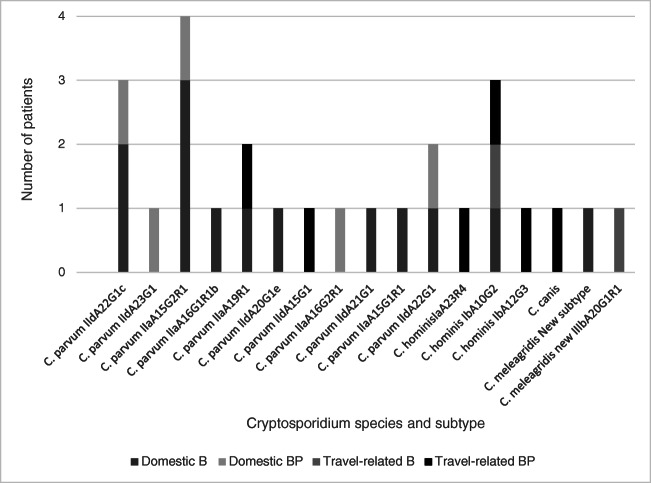
Results of molecular typing of Cryptosporidium species and subtype and suspected origin of transmission in the different patient groups

In total, 26 out of the 29 *Cryptosporidium* spp. were sent for species- and subtyping at the Public Health Agency of Sweden. The results and suspected origin of transmission are shown in Fig. 2. In group B, 14 cases were domestic and two travel-related. In group BP, six cases were domestic and seven travel-related. The positive samples related to travel came from Jordan (*C. canis*), Serbia, Philippines and Colombia (*C. hominis*), Spain and India (*C. parvum*), and Sri Lanka (*Cryptosporidium meleagridis* (*C. meleagridis*)).

Of the Cryptosporidium cases, 55% were female and 45% male. For *G. intestinalis*, 53% were male and 47% female. The age distribution of the positive cases of *Cryptosporidium* spp and *G. intestinalis* is shown in Fig. 1. The number of positive *E. histolytica* and *C. cayetanensis* was too few to evaluate statistically.

## Discussion

In the present study, we compared detection of protozoa in patients presenting with gastrointestinal symptoms, in patients with a suspicion of protozoa infection, in patients with suspicion of bacterial GE, and in patients with suspicion of both protozoa and bacterial GE. More than half of Cryptosporidium cases were detected in samples with no suspicion of parasite infection, but with a suspicion of bacterial GE. A recent study from Denmark showed that Cryptosporidiosis in children was under-recognized, and the prevalence was twice as high in patients with diarrhea without suspicion of parasite infection compared to those with a suspected parasite infection [3].

The differences in detection between the patient groups cannot be explained by differences in either distribution of age or sex since there was no statistically significant difference between the groups. The unrecognized cases of Cryptosporidium (patients in group B) were mostly domestic (14 out of 16 cases) compared to six domestic and seven travel-related in the BP group. This can be due to a misconception that parasites are not common in Sweden. It might also be because the clinical presentation of cryptosporidiosis is not associated with the common conception of parasite infections, which may be more associated with mild and long-lasting diffuse symptoms and not acute GE that Cryptosporidium can cause. No cases of Cryptosporidiosis was detected in group P, where only parasites were suspected, supporting the thought that cryptosporidiosis does not present clinically as a suspected parasite infection. As this study shows, Cryptosporidium is not an uncommon or unusual cause of GE in Sweden and the outbreak potential, and its ability to cause severe disease in immunocompromised patients should lead to a higher awareness especially in cases of domestic infections. In the present study, Cryptosporidium was the second most detected pathogen (viral agents were not included), after Campylobacter both in the group where only bacterial GE was suspected, and in all groups combined (same number of cases in total as *G. intestinalis*).

Almost all domestic Cryptosporidium cases were *C. parvum*. This finding is consistent with other studies, showing that *C. parvum* is the dominant species in Sweden [26]. There were ten different subtypes of *C. parvum* detected, three different *C. hominis*, and two *C. meleagridis*. *C. canis* was not subtyped since the gp60 gene of *C. canis* is yet not characterized and no primers are available. The most commonly detected subtypes of *C. parvum* was IIaA15G2R1 and IIdA22G1. Both are common subtypes in Europe, both in young cattle and in human cases [2, 5, 27].

Cryptosporidiosis was most common in the age group 21–40, which is consistent with national notifications data [9]. In the present study, one case in the age group 5–10 was detected. In other studies and in national notifications, children have had higher detection rates [2, 9, 28]. Perhaps there can be a seasonal reason, that more children are affected during the warmer months when there are more swimming and outdoor activities including animal contact, all of which are described as risk factors [27].

In the present study, two microscopy-positive Cryptosporidium cases were negative in PCR. One was a *C. canis*, and the primers and probe used do not allow detection of this type. The other was a *C. hominis* case, and even though the sample was run multiple times, it remained negative (inhibition control was positive). Many different species have been detected in Swedish patients, and a proper design of primers and probes used is of importance for ensuring detection [29–31].

Microscopy for Cryptosporidium is demanding both in time and in labor, but it can be done with a high sensitivity if it is actively searched for in all samples in wet mounts, together with an algorithm that uses staining with Ziehl-Neelsen based on symptoms and not specific request [20]. In this study, there was no statistically significant difference in detection between microscopy and real-time PCR in the two groups in which all samples were tested with both methods. In other studies, real-time PCR has increased detection compared to microscopy [32–34].

A group of blood donors was included as a control group to evaluate detection in healthy individuals. No cases of Cryptosporidium were detected in the control group. The size of the control group is a limitation to the study. This study shows nothing about the occurrence of cryptosporidiosis in healthy children or elderly, but in the age group 21–60, there was a statistically significantly higher detection in the patient groups B and BP (*p* = 0.03) compared to the control group. Cryptosporidium is a well-recognized significant pathogen [2] and is not shown to have a high prevalence in healthy people.

The two cases of *C. cayetanensis* were from group B and would routinely only have been tested for bacterial agents. Since the detection rate was so low, no conclusions can be drawn, but *C. cayetanensis* has increased in, for example, the USA [16], and attention should be given to a potential increase in other places.

One of the two cases of *E. histolytica* was detected in the B group. There are very few cases of *E. histolytica* in Region Jönköping County, and statistically, no conclusions can be drawn. However, it shows that there is risk of under-recognition which can lead to complications for patients.

Most cases of *G. intestinalis* were detected in groups BP or P. The three cases detected in the B group were domestic; in the P and BP groups, most patients were travel related. *G. intestinalis* seem to, in contrast to Cryptosporidium, have a clinical presentation that most often leads to suspicion of parasite infection, and also it is often detected in screening of (sometimes asymptomatic) immigrants. One case was also detected in the blood donor group from a donor with no history of gastrointestinal symptoms. Asymptomatic carriage of *G. intestinalis* can be common [35, 36], but *G. intestinalis* can also cause severe symptoms [11, 36]. A larger control group would have been better to determine asymptomatic infections in Swedish patients.

The present study only investigated samples from patients from one region which limits the generalization of the results; however, the results are likely applicable in more parts of Sweden and in countries with a similar testing regime [2, 3]. The high incidence in the two Swedish counties with a testing regimen to test all patients with diarrhea and the results of the present study show that there is likely an under recognition of protozoan infections, especially Cryptosporidium in Sweden. The clinical presentation of cryptosporidiosis is very similar to the infections caused by bacteria, and to distinguish the agent is not possible based solely on symptoms. New technologies present new possibilities, and the use of real-time PCR in multiplex assays offers the advantage of the possibility to combine detection of different types of agents (protozoa, bacterial, and viral) with similar clinical presentation in the same assay and sample.

It is possible that the bacterial agents were underdiagnosed with culture compared to molecular methods, and it was not part of this study to compare methods for bacterial detection. The blood donors were not tested for bacterial agents since the aim of the study was to investigate the incidence of diarrhea causing protozoa. The patients from the P group were not tested for bacterial agents due to the nature of sample; a fixed sample cannot be cultured.

In conclusion, cryptosporidiosis in Swedish patients was under-recognized, and there is a risk of under-recognition of more uncommon *E. histolytica* and *C. cayetanensis*. Diarrhea caused by these protozoa is likely more common than hitherto estimated in Sweden. In order to prevent both complications in immunocompromised patients and for faster detection of outbreaks, an accurate diagnosis is important. To switch to molecular detection is one factor in increasing detection, but the testing algorithm is likely as essential. A more symptom-based diagnostic regime will most likely increase detection and knowledge of these protozoan infections in Sweden.
